# Lost in translation: Inconvenient truths on the utility of mouse models in Alzheimer’s disease research

**DOI:** 10.7554/eLife.90633

**Published:** 2024-09-27

**Authors:** Alberto Granzotto, Bryce Vissel, Stefano L Sensi

**Affiliations:** 1 Center for Advanced Studies and Technology – CAST, University G. d’Annunzio of Chieti-Pescara Chieti Italy; 2 Department of Neuroscience, Imaging, and Clinical Sciences, University G. d’Annunzio of Chieti-Pescara Chieti Italy; 3 St Vincent’s Hospital Centre for Applied Medical Research, St Vincent’s Hospital Darlinghurst Australia; 4 School of Clinical Medicine, UNSW Medicine & Health, St Vincent's Healthcare Clinical Campus, Faculty of Medicine and Health, UNSW Sydney Sydney Australia; 5 Institute for Advanced Biomedical Technologies – ITAB, University G. d’Annunzio of Chieti-Pescara Chieti Italy; 6 Institute of Neurology, SS Annunziata University Hospital, University G. d’Annunzio of Chieti-Pescara Chieti Italy; University of Oxford United Kingdom; University of Oxford United Kingdom

**Keywords:** Amyloid, Tau, APOE, aging, neuroinflammation, neurodegeneration

## Abstract

The recent, controversial approval of antibody-based treatments for Alzheimer’s disease (AD) is fueling a heated debate on the molecular determinants of this condition. The discussion should also incorporate a critical revision of the limitations of preclinical mouse models in advancing our understanding of AD. We critically discuss the limitations of animal models, stressing the need for careful consideration of how experiments are designed and results interpreted. We identify the shortcomings of AD models to recapitulate the complexity of the human disease. We dissect these issues at the quantitative, qualitative, temporal, and context-dependent levels. We argue that these models are based on the oversimplistic assumptions proposed by the amyloid cascade hypothesis (ACH) of AD and fail to account for the multifactorial nature of the condition. By shedding light on the constraints of current experimental tools, this review aims to foster the development and implementation of more clinically relevant tools. While we do not rule out a role for preclinical models, we call for alternative approaches to be explored and, most importantly, for a re-evaluation of the ACH.

## Introduction

The accumulating failures of so many AD clinical trials, along with the recent, highly controversial Food and Drug Administration (FDA) approval of monoclonal antibodies – that at best show limited clinical benefits (Høilund-Carlsen et al., 2024; Kepp et al., 2023a) – provide reason to reconsider the molecular determinants of AD and the ACH, in particular.

In its original formulation, the ACH identifies the dysmetabolism of β-amyloid (Aβ) and its parenchymal deposition into senile plaques as the primary driver of a pathogenic, although still unclear, series of molecular events leading to the formation of hyperphosphorylated tau inclusions and, eventually, neuronal death (Hardy and Higgins, 1992). The non-linear association between Aβ plaques and cognitive deficits has led to revisions of the ACH, including the suggestion that soluble, low-molecular-weight Aβ oligomers – rather than plaques – are the primary neurotoxic species (Cline et al., 2018; Lambert et al., 1998), although this too remains highly controversial (Morris et al., 2018; Morris et al., 2014). The ACH is also at the foundation of the current ‘ATN research framework’ for AD. The ATN is designed to provide a structured and unbiased categorization of the AD continuum and is based upon biological/molecular changes identified by post-mortem examination or by biomarkers (i.e. amyloid – ‘A,’ tau – ‘T,’ and neurodegeneration – ‘N’ Jack et al., 2018). There continue to be good reasons to consider that the ATN construct offers a suboptimal and incomplete heuristic value (Morris et al., 2018).

While few scientists rule out any role of amyloid in the disease, there is growing skepticism around the sole centrality of Aβ in AD. Novel hypotheses are reconsidering the construct in the light of mounting discrepancies with recent clinical, epidemiological, and pharmacological findings (Granzotto and Sensi, 2024; Herrup, 2022; Herrup, 2015; Kepp et al., 2023a; Kepp et al., 2023b; Kurkinen, 2023; Liu et al., 2023; Morris et al., 2018; Morris et al., 2014). These discrepancies include but are not limited to, the large proportion of cognitively unimpaired elderly who have amyloid pathology, the long-known evidence for the contribution of mixed neuropathology in AD cases, and the modest benefits offered by Aβ-lowering antibodies [reviewed in Høilund-Carlsen et al., 2023, Granzotto and Sensi, 2024, and Guo et al., 2024]. In this context, two prominent studies investigating the effect of Aβ passive immunotherapy in cognitively unimpaired subjects at risk of developing AD challenged the validity of the ACH. The Alzheimer’s prevention initiative (API) Colombia study enrolled carriers of a mutation (PSEN E280A), conferring a particular risk of developing AD (Alzforum, 2022). The Anti-Amyloid Treatment in Asymptomatic Alzheimer’s Disease (A4) study enrolled subjects with elevated brain levels of Aβ as assessed by Amyloid PET imaging (Sperling et al., 2023). Both trials were unsuccessful, joining the long list of Aβ-targeting interventions that failed to produce clinically relevant benefits (Panza et al., 2019). As always, the amyloid proponents suggest it is not due to fallacies in the ACH and suggest other reasons for these trials' failure. However, these protestations are increasingly debated. Recently, Frisoni et al. attempted to reconcile the inconsistencies and proposed a more sophisticated view of the ACH that includes the contribution of stochastic elements to AD etiology, like environmental/modifiable factors and low-risk genes (Frisoni et al., 2022). However, the revised version still maintains a central role for amyloid in the disease. We do not exclude a role for amyloid. However, all current evidence points to an urgent need to begin revisiting the model, allowing for the likely molecular and cellular mechanisms, driven by a complex range of factors that ultimately generate Alzheimer’s dementia.

The above concerns call for an urgent revaluation of the ACH and the development of new hypotheses. The reappraisal of the ACH should also encompass a re-evaluation of the preclinical models designed to recapitulate the AD phenotype and frequently employed as tools for early safety/efficacy testing of drug candidates and for the identification of novel, druggable targets (Ganesan et al., 2024; LaFerla and Green, 2012; Scearce-Levie et al., 2020). Transgenic mammals, particularly mice, are the organisms of choice for investigating AD-related mechanisms in a complex in vivo setting. AD is also modeled on invertebrates, like *Drosophila melanogaster* and *Caenorhabditis elegans*. However, the phylogenetic distance from mammals of these systems limits their relevance and overall implications for the dementia field (Elder et al., 2010).

So far, more than 210 rodent models have been generated to recapitulate AD’s clinical features in research laboratories. Extensive, ongoing efforts have been and are made to engineer and characterize animal models to dissect the molecular mechanisms of the disease (Alzforum, 2023a). Unfortunately, the translational outcomes of these endeavors have, to date, been poor. We suggest that part of the reason is likely because of technical and biological limitations and, in some cases, conceptual flaws.

Differences in genetic background, transgenes, breeding and handling strategies, housing conditions, protocols for quantifying phenotypic traits, and endless additional variables make it challenging, if not impossible, to frame the information generated through these models within a consistent and comprehensive picture. Some of these issues have been discussed elsewhere (Errington, 2024; Mullane and Williams, 2019; Padmanabhan and Götz, 2023; Reynolds, 2022). Here, we will focus on the transgenic mouse models' shortcomings in terms of why they offer limited support for the ACH and why their use as preclinical models needs to be taken with caution (Table 1).

**Table 1.** Most common first- and second-generation transgenic models of Alzheimer’s disease (AD).

	Mouse line	Transgene(s)	Ref(s)
**First-generation** **APP transgenic mice**	PDAPP	APP V717F (Indiana)	Games et al., 1995; Rockenstein et al., 1995
	Tg2576	APP K670N, M671L (Swedish)	Hsiao et al., 1996
	APP23	APP K670N, M671L (Swedish)	Kelly et al., 2003; Van Dam et al., 2003
	J20	APP K670N, M671L (Swedish), V717F (Indiana)	Mucke et al., 2000
	TgCRND8	APP K670N, M671L (Swedish), V717F (Indiana)	Chishti et al., 2001
**APP and PSEN transgenic mice**	APPPS1	APP K670N, M671L (Swedish); PSEN1 L166P	Radde et al., 2006
	5xFAD	APP K670N, M671L (Swedish), I716V (Florida), and V717I (London); PSEN1 M146L and L286V	Oakley et al., 2006; Tang et al., 2016
**Second-generation knock-in APP transgenic mice**	App knock-in (humanized Aβ)	App G676R, F681Y, R684H (humanized Aβ)	Serneels et al., 2020
	APP^NL-F^	Humanized Aβ+APP K670N, M671L (Swedish), I716F (Iberian)	Saito et al., 2014
	APP^NL-G-F^	Humanized Aβ+APP K670N, M671L (Swedish), I716F (Iberian), E693G (Arctic)	
	APP^SAA^	Humanized Aβ+APP K670N, M671L (Swedish), E693G (Arctic), T714I (Austrian)	Xia et al., 2022

In the following sections, we will summarize the limitations in the preclinical modeling of AD that are sketched – in broad brush strokes – at the *qualitative*, *quantitative*, *temporal*, and *context*-dependent levels. However, we also discuss the opportunities offered by mouse models for addressing some still unresolved scientific questions.

### Qualitative

#### Mice with APP and PSEN mutations

**Figure 1.**
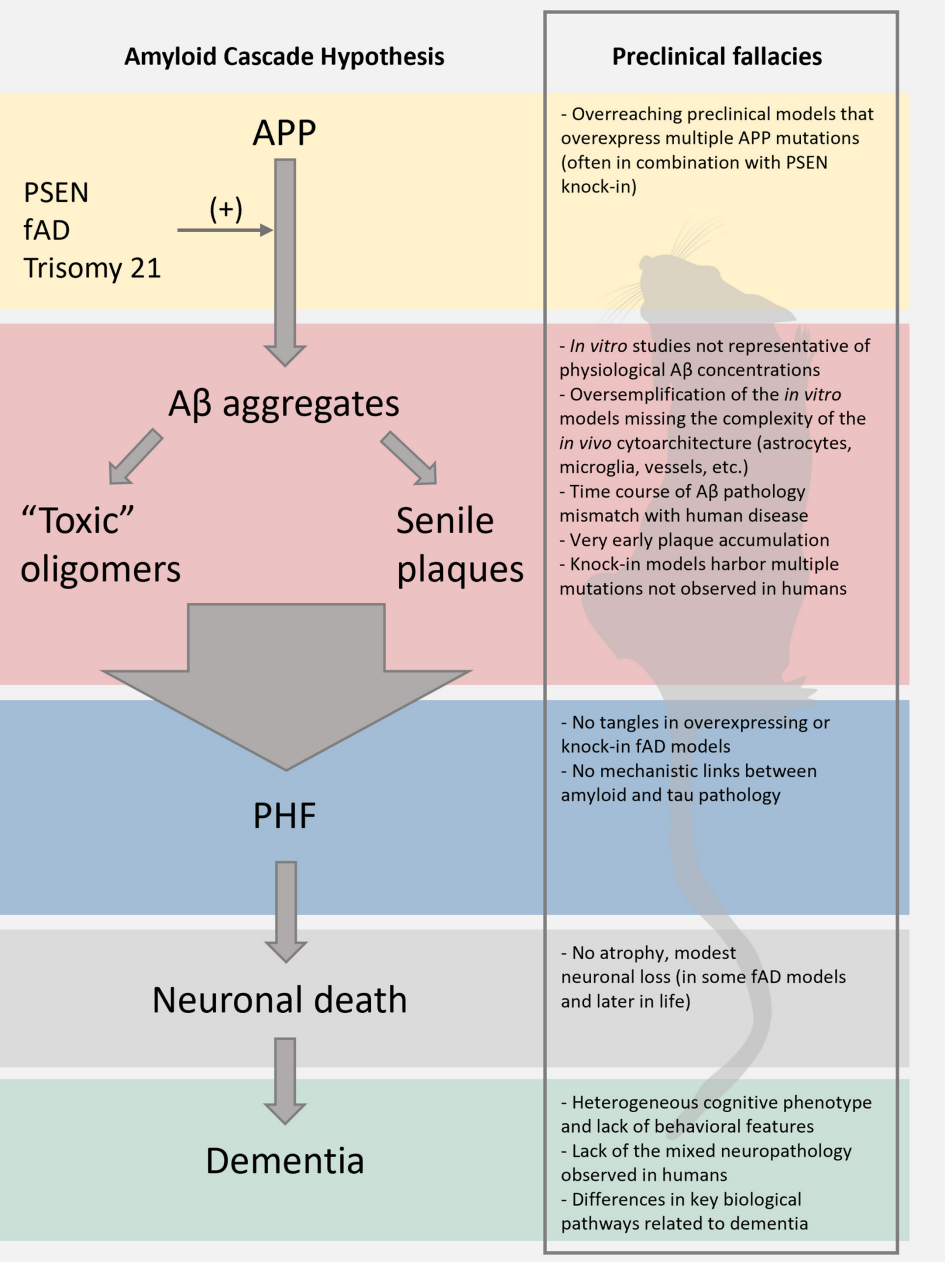
Limitations of the preclinical mouse models of Alzheimer’s disease (AD). The scheme reports the pillars of the amyloid cascade hypothesis (ACH) left; modified from Karran et al., 2011. For each step, we aimed at identifying key limitations in the preclinical modeling of the cascade. We envision that these pitfalls, along with discrepancies of the amyloid construct cascade itself, critically dampen the potential translational value of these models.

Most preclinical studies employ transgenic rodent models that, to different degrees, express human genes whose mutations are associated with the familial form of AD (fAD). Mutations in *PSEN1* [encoding presenilin 1 (PSEN1)], *PSEN2* [encoding presenilin 2 (PSEN2)], and/or *APP* [encoding amyloid precursor protein (APP)] affect APP processing and are causally implicated in the development of autosomal dominant AD (Figure 1). In many cases, to drive any phenotype, the mice express more than one of such mutations. However, the very low prevalence of fAD – less than 1% of total cases (Pavisic et al., 2020) – makes the findings obtained in these models impossible to generalize to the broad spectrum of sporadic AD cases (sAD), causing misleading overinterpretation of the results.

Long-term monitoring of neuropathological and functional changes in these models reveals a composite scenario that often conflicts with the clinical features of sporadic sAD (Drummond and Wisniewski, 2017). Two common issues with fAD models that contrast with the clinical manifestations of human disease are the absence of tau pathology and brain atrophy. Most models do not show neurodegeneration. When neurodegeneration does occur, modest neuronal loss is observed, and it is usually confined to discrete brain regions (e.g. a single layer of the cortex or hippocampal subfields) and even then, only in a small fraction of amyloid-dependent models of AD. Of note, these minor signs of neurodegeneration are primarily described in transgenic mice in which *APP* mutations occur in the presence of *PSEN1* mutations, thereby raising questions on the specific contribution of Aβ versus the role of *PSEN1* in causing the pathological phenotype. Indeed, early studies pinpoint *PSEN* mutations in the mouse models as the likely primary drivers of features of AD-related neuronal dysfunction, like dysregulation of calcium (Ca^2+^) signaling, metal ion dyshomeostasis, synaptic dysfunction, impaired adult neurogenesis, and increased neuronal vulnerability to cytotoxic stimuli (Al Rahim et al., 2020; Corona et al., 2011; Duff et al., 1996; Hernandez-Sapiens et al., 2022; Mattson et al., 2000; Stutzmann et al., 2006; Stutzmann et al., 2004). Although not systematically studied, the effect of PSEN1 in animal models seems to occur in an Aβ-pathology independent fashion, as mice harboring *APP* and *PSEN1* fAD mutations lose their phenotype upon removal of the *PSEN1* mutation. Conversely, single *Psen1* KI mutants continued to display functional alterations (Bomba et al., 2013; Stutzmann et al., 2006). Overall, this evidence suggests a critical role for *PSEN* in neurological function, a construct that has been conceptualized in the ‘presenilin hypothesis of AD’ (Shen and Kelleher, 2007; Yan et al., 2024). The construct identifies *PSEN* mutations and/or impaired PSEN functioning as the primary contributor to neurodegeneration in fAD. Importantly, the presenilin hypothesis offers an alternative view of fAD pathogenesis (Kelleher and Shen, 2017) as, contrary to the ACH, it points to the accumulation of Aβ as the byproduct of a faulty enzymatic activity and not as the trigger of AD per se. Notably, systematic analysis of fAD-causing *PSEN1* mutations has shown that in 75% of the dementia cases, the mutations led to decreased production of Aβ fragments (Sun et al., 2017), supporting the notion that amyloid is not the disease driver. Conversely, it is not out of the question that human AD-causing mutations in APP contribute to AD via an effect on presenilin function. Thus, if the mouse models harboring PSEN (or PSEN + APP) mutations offer value, they may be a model of the presenilin hypothesis rather than a model of the ACH.

The idea that PSEN dysfunction – rather than Aβ – is central to AD development is further supported by the clinical failures of γ-secretase inhibitors. γ-secretase comprises several subunits, including PSEN, and – beyond the cleavage of APP – the enzyme has numerous roles in the central nervous system (CNS). Unsurprisingly, γ-secretase inhibitors, developed to limit APP cleavage and amyloid production, have been consistently found to worsen cognition in clinical trials (Coric et al., 2015; Doody et al., 2013). The effect was likely attributed to the inhibition of PSENs activity and its negative downstream impact on multiple signaling pathways, like Notch signaling (Hur, 2022).

The topological distribution and the mechanisms of – albeit modest – neuronal loss of these fAD models offer additional inconsistencies with the ACH (Jankowsky and Zheng, 2017). In these transgenic mice, neuronal demise, when present, often occurs in the form of necrotic cell death near senile plaques, a finding more in line with ‘mechanical’ disruption of neuronal integrity rather than the result of a chronic, staged, and regulated process of neuronal dysfunction (Tanaka et al., 2020). There are, of course, a few exceptions, such as the J20 model (Wright et al., 2013).

To circumvent the drawbacks posed by the first-generation overexpressing models, researchers have developed second-generation knock-in transgenic mice in which the *App* gene is humanized with the addition of fAD mutations. These models exhibit alterations of Aβ metabolism that precede subtle cognitive deficits, Aβ_42_ overproduction, increased Aβ_42_/Aβ_40_ ratio, and neuroinflammation without the pitfalls associated with *APP* overexpression. Although the approach bypasses the limitations posed by first-generation mice, critical drawbacks and divergence with clinical observations remain. For instance, to produce Aβ neuropathology, knock-in models require the presence of multiple *APP* mutations not found in humans. The *App^NL^* mice, a model that carries only the ‘pathogenic’ Swedish mutation, failed to develop amyloid pathology up to 22 months of age (Saito et al., 2014). Surprisingly, *App^NL^* mice are proposed as a negative control for the multiple *App* knock-in strains (Alzforum, 2023b; Saito et al., 2014). Notably, assessment of synaptic functioning in *App* knock-in mice revealed only presynaptic alterations and not the postsynaptic alterations seen in humans. This suggests again that other factors like presenilins or gross inflammation, rather than a direct action of Aβ per se, may cause human post-synaptic dysfunction and neuronal loss (Benitez et al., 2021). Similarly, the novel *App* knock-in mouse model *App^SAA^* harbors multiple disease-causing mutations (Swedish, Arctic, and Austrian) to promote Aβ pathology. Surprisingly, and in contrast to AD patients, *App^SAA^* mice exhibit increased brain metabolism (measured by FDG-PET) as Aβ pathology progresses (Xia et al., 2022). Like the first-generation models, knock-in mice do not develop tau pathology nor generate overt signs of neurodegeneration.

#### Mice with tau mutations

Several mouse lines have been engineered to mimic the inclusions of hyperphosphorylated tau observed in AD patients, which may potentially overcome the lack of tau pathology of APP strains. Tau pathology is considered an accurate correlate of AD-related neurodegeneration, as the extent and topological distribution of tau accumulation mirrors the disease’s clinical course more faithfully than other biomarkers (Knopman et al., 2021). While they are often considered models of AD, most tau models overexpress the human *MAPT* gene, harboring mutations absent in AD cases but associated with frontotemporal lobar degeneration (FTLD). Unlike APP models, these mice better phenocopy some of the clinical features of AD, like neurofibrillary tangles (NFT) inclusions, neurodegeneration, and cognitive deficits. However, the strong genetic drive required to display an overt tau pathology raises questions on the generalizability of the findings when applied to sporadic forms of tauopathies, including AD.

An additional caveat is that tau isoforms differ between humans and mice. In humans, alternative splicing of the *MAPT* gene gives rise to six tau isoforms characterized by differences in length, N-terminal sequences, and the presence of three- (3 R) or four- (4 R) repeated microtubule-binding sequences (Hernández et al., 2020). Compared to the human homolog, murine tau differs in terms of the number of repeats (tau 3 R is absent in adult mice) as well as in the sequence of the N-terminal domain (11 amino acids shorter in mice) (Hernández et al., 2020). These features might be critical for shaping the physiological and pathological properties of the protein. For instance, the N-terminal domain is relevant for the tau-driven modulation of proteins involved in neuronal functioning (i.e. NMDA receptors Miyamoto et al., 2017, Synapsin-1, and Synaptotagmin-1, among others Hernández et al., 2020; Stefanoska et al., 2018). Changes in the ratio between 3 R and 4 R isoforms are also different from what is found in neurodegenerative tauopathies, including AD (Bowles et al., 2022; Cherry et al., 2021; Ginsberg et al., 2006). Therefore, substantial over-expression of the human form of tau in mice, an experimental setting in which tau and its binding partners are profoundly different, may result in potential artifacts and findings with poor translational value. Notably, recent findings concerning immune-mediated neurodegeneration in animal models suggest new mechanisms of degeneration in human tauopathies (Chen et al., 2023). If the evidence continues to stack up, immune dysfunction in the tau mice may become a model of disease, at the very least for tauopathy, and could be worth pursuing.

#### Mice with multiple transgenes

Age-dependent Aβ accumulation is common to many non-human species (i.e. non-human primates, dogs, sheep). However, whether this impacts animal cognition remains unclear, with the neuropathological features of AD mostly a matter for human beings. No other non-human animal – except possibly the *Octodon degus* (Steffen et al., 2016) – displays the coexistence of Aβ pathology, NFT inclusions, glucose dysmetabolism, and neurodegeneration (Walker and Jucker, 2017). To generate a more robust phenotype, transgenic models harboring mutations on the *APP* and/or *PSEN* and the *MAPT* gene have been developed, like the 3xTg-AD or the TauPS2APP (Grueninger et al., 2010; Oddo et al., 2003). These examples indicate that extreme measures are required to generate models encompassing some of the critical features of AD. This is in stark contrast to humans, where the vast majority of AD cases occur without these gene mutations.

The failure of APP mutants to recapitulate disease without human tau mutations may, at the very least, point to the possible importance of crosstalk between Aβ and tau. *Mapt* knock-out mice crossbred with *APP* transgenic models show reduced neuronal deficits and improved memory performance compared to mice harboring the wild-type *Mapt* gene. These results suggest that tau confers toxicity to Aβ and not vice versa (Roberson et al., 2007; Ittner et al., 2010; Sasaguri et al., 2017). In addition, recently generated double knock-in mice harboring all six human MAPT isoforms and the humanized *App^NL-F^* gene have been characterized. Notably, the humanization of the murine tau gene was found sufficient to accelerate the propagation of pathological tau independently of the presence of Aβ (Saito et al., 2019). These observations suggest that tau in fact may sit above Aβ in the cascade of events leading to AD.

### Quantitative

In vitro and in vivo studies are primarily performed in settings in which Aβ concentrations are several orders of magnitude above the physiological range (Figure 1). In vitro evidence, designed to demonstrate the neurotoxic properties of synthetic Aβ adducts – in their different lengths and flavors –, was based on cultured neurons exposed to nanomolar concentrations of low-molecular-weight oligomeric forms of the peptide. These levels are a thousand-fold higher than the concentration found in vivo, usually in the picomolar range (Kepp et al., 2023a). The physiological relevance of such high Aβ concentrations is dubious. Mounting evidence suggests that physiological Aβ levels exert neurotrophic-like effects on synaptogenesis, neuronal survival, growth, and differentiation (Giuffrida et al., 2009; Yankner et al., 1990; Zhou et al., 2022). This dichotomic behavior (where low concentrations have opposite effects than high concentrations) is common to many molecules, like for instance, neurotrophins where the balance between proBDNF and mature BDNF levels acts on the opposite side of the neurodegenerative-plasticity spectrum (Brem and Sensi, 2018).

A further, often neglected issue of in vitro and in vivo studies using Aβ oligomers is the lack of control over the aggregation state of Aβ. The in vitro and in vivo extracellular milieu contains variable amounts of molecules and ions that are known to affect Aβ conformation (i.e., proteins, oxidizing and reducing agents, metal ions, and cell released Aβ cleaving enzymes). Finally, two independent studies reported that Aβ dimers – long considered central species in amyloid-driven toxicity – may be artifacts driven by SDS-based sample processing (Pujol-Pina et al., 2015; Watt et al., 2013).

Other quantitative pitfalls are associated with in vivo genetic models of AD. Three key aminoacid substitutions make murine Aβ less prone to aggregation when compared to its human counterpart. To overcome this issue, first-generation transgenic models overexpressed variable copy numbers of the *APP* gene harboring different AD-related mutations (Figure 1). The approach successfully generated Aβ-enriched plaques in the brain of the transgenic models. Yet, several limitations and questions of the relevance to human AD remain:

1. Unlike the mice, AD does not appear to involve overexpression of the entire *APP* gene (Harrison et al., 1996; Matsui et al., 2007), which can per se be harmful to neuronal functioning, eventually resulting in cytotoxicity (Bartley et al., 2012; Benitez et al., 2021; Bolognesi and Lehner, 2018).
2. Overexpression of APP includes other fragments besides Aβ, whose role is still largely underexplored. It remains possible that other fragments of APP drive toxicity. Indeed, it has never been ruled out that changes to the expression of the C99 fragment of APP underlie the neurodegeneration of fAD, as initially noted by John Hardy in his original paper on the ACH as an alternative mechanism of fAD (Hardy and Higgins, 1992).
3. Not all the *APP* mutations linked to fAD are consistently associated with Aβ overproduction. While some ‘pathogenic’ mutations, like the Swedish (K670N/M671L), Flemish (A692G), or London (V717I), increase Aβ production, others like the Italian (E693K), the Dutch (E693Q), the Arctic (E693G), or the Osaka (E693Δ) mutations produce unaltered or even reduced levels of Aβ fragments (De Jonghe et al., 1998; Nilsberth et al., 2001; Tiwari and Kepp, 2016).
4. The biological significance of the Aβ_42_/ Aβ_40_ ratio – a widely employed biomarker of brain Aβ deposition – is debated (Imbimbo et al., 2023; Kepp et al., 2023b).
5. Overexpression might be toxic per se by disrupting other genes in the proximity of the insertion site of the transgene or by engulfing cellular proteostasis (Alzforum, 2023c; Saito et al., 2016; Saito et al., 2014).
6. Some of the phenotypes observed in first-generation AD models can also be critically reconsidered in light of the ‘presenilin hypothesis of AD’ as the increased workload of PSENs to metabolize overexpressed APP may divert the enzyme from the cleavage activity of the many other physiologically relevant substrates required for neuronal functioning (Haapasalo and Kovacs, 2011).

As above, the transgenic animal models drive very high levels of APP production, with the effect that high concentrations of Aβ are generated. Other approaches involve the injection of Aβ oligomers directly into the rodent brain. Both strategies can model some of the key effects of the disease, such as synapse loss. However, it is also likely that the high, unnatural monomer or oligomer concentrations drive additional responses, such as activation of inflammatory responses that per se produce damage in an amyloid-independent fashion.

‘Quantitative’ concerns also apply to tau models of AD. Although high levels of total tau have been reported in AD patients, there is no consensus that tau overexpression occurs in AD (Hier et al., 2022). The PS19 and the rTg4510 are two of the most widely used models of tau pathology for AD. They harbor the human 4 R tau with the P301S and the P301L mutation, respectively. However, these models generate expression levels that are 5- (for PS19) to 13-fold (for rTg4510) higher than the endogenous murine tau (Jankowsky and Zheng, 2017). The results are early signs of tau hyperphosphorylation, NFTs formation, neurodegeneration, overt cognitive and motor deficits, and early lethality (Lewis et al., 2000). Similar traits, however, have been reported in mice overexpressing wild-type murine tau. These findings thereby indicate that, in mice, tau overexpression is sufficient to promote neurotoxicity independently of the tau genotype (Adams et al., 2009).

#### Temporal

Despite decades of research efforts, aging remains the primary risk factor for AD (Herrup, 2010; Mattson and Arumugam, 2018). Aging offers the ideal battleground where multiple molecular determinants could wreak havoc in the brain. This aspect has not been adequately considered as a cofactor in the preclinical modeling of the disease or the critical interpretation of the results (Padmanabhan and Götz, 2023). The time-dependent loss of physiological fitness impinges on many of the very same mechanisms linked to AD pathogenesis, like oxidative stress, mitochondrial dysfunction, impaired DNA repair, altered cellular metabolism, ion dyshomeostasis, aberrant neuronal network functioning, neuroinflammation, vascular disease, senescence, and stem cell exhaustion (López-Otín et al., 2013; Mattson and Arumugam, 2018). The gist is: is the dysregulation of Aβ and tau that accelerates cellular demise during aging, or is a yet unidentified perturbation of the trajectory of physiological aging that results in the accumulation of misfolded proteins as a byproduct?

**Figure 2.**
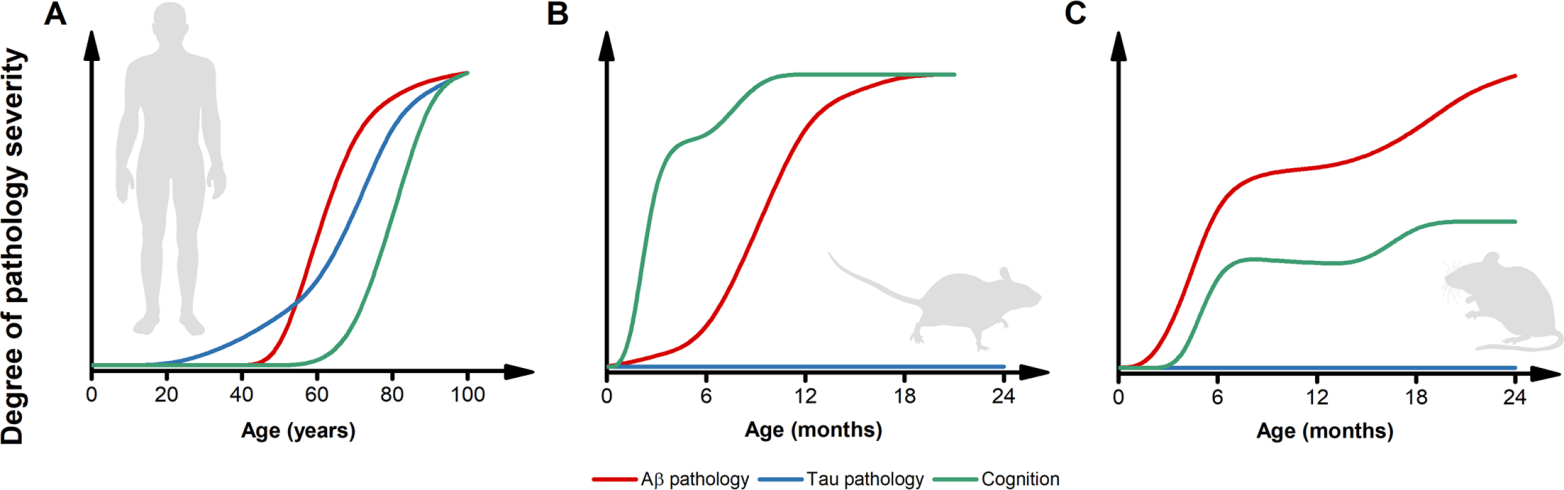
Inconsistencies in the trajectories of Alzheimer’s disease (AD) pathology between humans and preclinical models. (**A**) The pictogram illustrates the dynamics of β-amyloid (Aβ) (red) and tau (blue) pathology as well as the trajectory of cognitive symptoms (green) in the sporadic forms of AD (modified from Frisoni et al., 2022). Please note that, in the case of familial form of AD (fAD) or APOEε4-related AD, the pathology follows a similar sequence of events but with early and steeper trajectories (Frisoni et al., 2022). (**B**) The pictogram estimates the dynamics of key AD features as observed in the most widely used AD mouse models. Unlike what is observed in humans, in these preclinical settings, cognitive deficits usually anticipate the appearance of Aβ pathology. Tau inclusions and signs of overt neurodegeneration are absent. (**C**) The pictogram estimates the dynamics of key AD features as observed in second-generation knock-in mouse models of AD. In this experimental setting, Aβ pathology anticipates the development of subtle cognitive decline (Sakakibara et al., 2018). Like first-generation overexpressing models, tau tangles and brain atrophy are absent. The trajectories in B and C have been estimated by employing data extracted from publications using the mouse models listed in Table 1 and normalized for each pathological feature. Time courses of the original reports were used whenever possible, alternatively, early studies investigating the time-dependent changes in the phenotype of these models were interrogated.

The early and aggressive presentation of amyloid- and tau-pathology observed in preclinical models of AD does not help to resolve this critical issue (Figure 2). Commonly used AD mouse models, like the 5xFAD, display amyloid deposits starting at 2–4 months of age (Oblak et al., 2021). The J20 mice develop amyloid pathology a bit later, following the onset of inflammation; however, this still occurs at a relatively young age (Wright et al., 2013). With all due limitations, this early accumulation can be translated to Aβ deposits occurring in 4–8 year-old humans, a scenario not found even in the most aggressive cases of fAD, let alone sAD. It is also worth noting that even fAD cases require decades for the disease to take hold, usually when carriers of *APP* or *PSEN* mutations are in their 40 s or 50 s (Frisoni et al., 2022). These observations suggest that (1) the human brain can cope for decades with the genetically driven accumulation of Aβ and/or that (2) additional age-related factors are required for disease onset. However, we acknowledge the argument that the purpose of the animal models is to accelerate pathology to study the disease, thereby requiring an aggressive phenotype to attempt to model human AD.

A temporal explanation is also called in support of the fact that first- and second-generation fAD models fail to develop tau pathology and brain atrophy. The case is frequently made that lack of NFT and overt neuronal loss rely on the AD time course since the short lifespan of rodents prevents the development of Aβ-driven tauopathy and neurodegeneration observed in humans. However, these arguments do not align with observations from non-human primates (Walker and Jucker, 2017). These mammals show steeper aging trajectories when compared to humans, with extensive cerebral Aβ deposition that occurs at ages proportional to their lifespan and in the absence of tau pathology or overt signs of dementia (Finch and Sapolsky, 1999; Walker and Jucker, 2017).

#### Context

The accumulated clinical evidence indicates that several genetic and environmental factors, with different potency, have been acknowledged as AD contributors (Frisoni et al., 2022; Knopman et al., 2021; Livingston et al., 2020).

In that context, molecular genetics analyses provide invaluable information on AD’s complex etiology. Besides the rare mutations on *APP*, *PSEN1*, and *PSEN2* genes showing an essentially 100% penetrance, linkage and genome-wide association studies (GWAS) have identified well over 20 additional genetic risk loci (Andrews et al., 2023). Although the contribution of each associated gene was frequently interpreted in relation to Aβ- or tau-pathology, it is notable that these genes belong to three major pathways: cholesterol and lipid metabolism, immune system and inflammatory responses, and endosomal vesicle cycling (Van Cauwenberghe et al., 2016).

In this context, the contribution of APOE is an area of active investigation (Chen et al., 2021; Ganesan et al., 2024). This key protein is involved in fat metabolism – including cholesterol. In humans, three major allelic variants exist: APOEε2, ε3, and ε4 (Huebbe and Rimbach, 2017). Each genotype is strongly associated with a different risk of developing the late-onset form of the disease with the ε4 isoform increasing it while the ε2 being protective. The most common ε3 allele is considered neutral (Serrano-Pozo et al., 2021). Given the central role played by APOE in cholesterol metabolism and AD, caution must be exercised when interpreting results from preclinical models, as substantial dissimilarities exist among species in this very specific pathway. First, important differences concern APOE itself. The single mouse APOE isoform (mAPOE) shares only 70% of the homology with its human counterparts. This is a serious red flag, considering that the three human isoforms, 299 amino acids long, differ from each other for just up to two residues (Frieden and Garai, 2012). In agreement, early studies comparing the effects on Aβ deposition of human isoforms vs. mAPOE revealed that mAPOE significantly accelerates plaque formation compared to its humanized homolog (Fagan et al., 2002). In addition, the daily turnover of the brain sterol pool is more than an order of magnitude higher in mice than in humans (0.4% vs 0.03% per day, respectively) (Dietschy and Turley, 2004). These findings indicate different synthesis, transport, and clearance needs that, in AD transgenic models, are likely to affect the pathology burden (Granzotto et al., 2011). Similarly, other risk factor-related genes for AD are often quite different in gene structure and processing in mice.

Profound metabolic changes also accompany AD. Epidemiological evidence indicates that metabolic alterations are strongly involved in AD pathogenesis, with obesity and diabetes being included in the list of the 12 modifiable risk factors that account for around 40% of all dementia cases (Livingston et al., 2020). In the brain, insulin acts as a potent neurotrophic factor where it modulates critical activities, like synaptic plasticity and cognitive functions (Arnold et al., 2018). Importantly, central insulin resistance and defective insulin signaling have been consistently observed in human post-mortem studies, leading to the hypothesis that AD is a ‘Type 3 diabetes’ (Steen et al., 2005). Obesity, a risk factor for diabetes, is also increasingly recognized as an active player in AD. Chronic inflammation associated with obesity contributes to neuroinflammation, and adipokines, bioactive molecules secreted by adipose tissue, may have neuroinflammatory and neurodegenerative effects (Bomba et al., 2019; Kotredes et al., 2023; Mooldijk et al., 2022). In this context, calculations estimate a sevenfold higher basal metabolic rate in mice *vs* humans (Terpstra, 2001), a difference that might affect pathology progression or, as demonstrated in other settings, influence the effectiveness of disease-modifying interventions (Gordon-Larsen et al., 2021; Terpstra, 2001).

Brain inflammation is emerging as a core feature of AD. The last few years have witnessed a significant advancement in our understanding of how inflammatory processes modulate the pathogenesis of AD (Kinney et al., 2018; Morris et al., 2018; Morris et al., 2014; Paolicelli et al., 2022). Robust associations were identified between AD susceptibility and genetic variants linked to genes specifically expressed by myeloid cells. These include *CD33*, *CLU*, *MS4A4A* and *MS4A6A*, *PLCG2*, *SORL1*, and *TREM2* (Andrews et al., 2023; McQuade and Blurton-Jones, 2019). Functionally, these genes largely encode proteins involved in phagocytosis, a central and therapeutically exploitable process in AD (Andrews et al., 2023). In parallel, novel research tools have disclosed an even more composite scenario (Hasselmann and Blurton-Jones, 2020; Paolicelli et al., 2022). Comparative single-cell analysis of humans vs mice showed that brain cells of the two species exhibit similar transcriptomic profiles in physiological settings, but remarkable changes occur upon pathological conditions (Zhou et al., 2020). The effect is particularly prominent in microglia, the immune cells of the brain (Zhou et al., 2020). A twofold interpretation can be drawn. Either the disease model in preclinical settings differs from the human AD, and/or the response to AD pathology is highly context- and species-dependent. In addition, growing evidence helped to profoundly revise the view of the brain as an immune-privileged organ (Louveau et al., 2015), with cells of the adaptive immune system and the peripheral-central immune crosstalk increasingly recognized with a causal role in the pathophysiology of AD (see Andrews et al., 2023; Bettcher et al., 2021; Haage and De Jager, 2022 for comprehensive reviews on the topic).

An additional context-dependent issue in AD modeling is posed by the heterogeneous set of neuropathology that, at the population level, contribute to dementia in older adults (Boyle et al., 2018; Brenowitz et al., 2017). Post-mortem data reveal that most aging brains are the target of mixed neuropathology (i.e. AD, cerebral amyloid angiopathy, TDP-43, Lewy body, atherosclerosis, etc.) (Boyle et al., 2018) while the isolated presence of senile plaques and NFTs is found only in a tiny fraction of dementia cases (Boyle et al., 2021; Boyle et al., 2018; Brenowitz et al., 2017; Morris et al., 2018). Post-mortem examination of >1000 dementia cases identified >230 different neuropathological combinations (Boyle et al., 2018), indicating almost person-specific pathological signatures and disease trajectories. This complexity cannot be recapitulated in preclinical settings.

When delving into AD, it is crucial to consider sex-related factors. The risk of developing AD is nearly double in women, a difference not fully explained by the female longer life expectancy (Reed-Geaghan, 2022). Although many studies are investigating sex-based differences in preclinical models, the results should be interpreted with caution. Indeed, biological differences can bias the outcomes. Among others, female mice lack the reproductive senescence features, including menopause and the extended post-reproductive periods, that characterize at least one-third of women’s lifespan and that fall within the most critical timeframe for developing early signs of dementia (Moir and Tanzi, 2019). In agreement, a causative role of the dysregulation of sex hormones in explaining the higher vulnerability of women to AD has been proposed (Carroll et al., 2007; Ratnakumar et al., 2019; Xiong et al., 2022).

For what concerns preclinical models, additional differences in the underlying biology of humans and mice need to be more carefully considered when modeling AD, testing interventions, and interpreting the data. For instance, nocturnal rodents have opposite circadian cycles when compared to humans. Since most experimental procedures are performed during the rodent inactive phase, recent findings suggested that circadian rhythms might influence and bias translational studies (Esposito et al., 2020). This might also occur in the context of AD, considering the importance of inactive phases (i.e. sleep hours) for the clearance of brain interstitial fluids from proteins and solutes accumulated during the wake/active cycles, like Aβ and tau (Holth et al., 2019; Roh et al., 2012). We raise this point to be comprehensive, but do not suggest it is the major limitation of the animal models.

Finally, the experimental conditions under which laboratory animals are typically housed often overlook the significant impact of an enriched environment, social engagement, physical activity, and natural pathogens or pollutants – all crucial factors observed in real-world scenarios – on the development and progression of AD (de Sousa et al., 2023; Dhana et al., 2024; Kivipelto et al., 2018). These environmental elements play a core role in influencing molecular mechanisms that promote brain resilience against age-related insults, like the activation of neurotrophic and bioenergetic signaling pathways, vital factors for maintaining cognitive health and mitigating the effects of neurodegeneration (Cotman and Berchtold, 2002; Mattson and Arumugam, 2018). Incorporating these environmental features within experimental models is essential for capturing the holistic impact of lifestyle elements on AD pathology and developing more translatable therapeutic strategies.

#### Implications for the ACH

First-generation of APP (or tau) overexpressing mouse models remain the primary tools in use for studying AD (Table 1). The original findings with these and similar AD model mice were considered strong support of the ACH. In hindsight, studies showing that overloading the brain from birth with foreign protein/s generates a phenotype, while its/their removal has some subsequent benefit, may have been over-interpreted in support of the ACH. The interpretation is problematic not only in the context of the qualitative, quantitative, temporal, and contextual limitations of the models as discussed herein but also because of the absence of adequate controls for extensive protein overload.

While the data do not exclude that Aβ and/or tau may contribute to dementia, additional risk factor-related genes (e.g. *TREM2*, *MS4A*, *CLU*, etc.) or environmental cues should be tested in enriched preclinical settings. Embracing the complexity of the molecular mechanisms of AD could substantially advance our comprehension of the disease and help therapeutic strategies.

#### Is all lost?

While a purely Aβ-centric view of dementia is no longer tenable (Granzotto and Sensi, 2024; Herrup, 2015; Kepp et al., 2023a; Morris et al., 2018; Morris et al., 2014), preclinical mouse models may still provide answers to disease-related questions in three primary respects.

First, mice are valuable for investigating the fundamental mechanisms through which perturbation of cellular interactions leads to brain dysfunction. Specifically, the commonly used mice – such as the J20 (Mucke et al., 2000; Wright et al., 2013) –, induced neuroinflammation or senescence models, as well as non-genetically modified animals, may prove helpful in studying the consequences of altered cellular interactions associated with inflammatory response and/or aging, two critical factors in dementia (Engelhart et al., 2004). While the trigger of inflammation in the mouse models, such as the J20 mouse model, namely the ectopic overexpression of human proteins, may not be identical to what drives human AD, these models are still valuable for further our understanding of the role of aberrant microglial astrocytic and adaptive immune responses in neuronal and synapse dysfunction.

Second, the commonly used mice expressing full-length mutant APP and/or mutant PSENs help unravel Aβ-independent mechanisms involved in AD. In other words, what is the role of the full human APP, the presenilins, and the various fragments and isoforms, beyond their effects on Aβ, in brain function and pathology? These mechanisms have been investigated (Saganich et al., 2006) but they remain greatly under-explored.

Third, a reconceptualization of murine models is needed. A question remains whether mice engineered with humanized genes (such as *APP*, *MAPT*, *APOEε4, TREM2,* and so on) will prove valuable for modeling human AD. Many of us are hopeful, but it is still unclear how biochemical and cellular signaling mechanisms in mice interact with human genes, an area in need of further consideration.

#### Alternative possibilities and future directions

Collective efforts are underway to develop better, more informative, and predictive models to improve translation from animal to humans (Vitek et al., 2020). These include the generation of mice combining multiple genetic and environmental risk factors (Ganesan et al., 2024; Rizzo et al., 2023) or the development of novel strains to evaluate the impact of naturally occurring genetic variations (limited in laboratory strains) on the AD phenotype (Neuner et al., 2019; Onos et al., 2019). In that respect, we are cautiously excited by the MODEL-AD project (MODEL-AD Consortium, 2024). The outcomes of these studies designed to generate sAD-relevant models could be highly informative (Kotredes et al., 2023). In addition, AD involves complex mechanisms beyond amyloid plaques and tau tangles, like synaptic dysfunction, mitochondrial impairment, Ca^2+^ dysregulation, neuroinflammation, oxidative stress, metal ion dyshomeostasis, and disruptions in neuronal signaling. As we noted above, there are also questions about the effects of the environment and natural pathogens on the brain, which are also not well modeled in pristine mouse facilities. Animal models often fail to fully replicate the complex interplay of the environment and the molecular and cellular processes that are strongly associated with disease symptoms. A better modeling and a deeper exploration of these mechanisms – alone or in synergy with the pathological features of AD – could inform the development of more targeted interventions.

It is also imperative to heighten methodological rigor in investigations employing murine models. The inadequacy of adhering to the rigorous standards observed in human clinical trials should be addressed. For instance, at a minimum, implementing blinding protocols and preferably blinding across all aspects of the analyses has become mandatory to generate data with translational value (Cozachenco et al., 2023). Regrettably, most animal studies do not consistently observe such practices (Errington, 2024; Reynolds, 2022).

There has been a notable shift towards human-centric approaches, emphasizing human-derived cellular models, organoids, and larger animals, like non-human primates. Human cells of the central nervous system, usually derived from induced pluripotent stem cells (iPSC), have emerged as indispensable tools to delve into disease mechanisms specific to human biology. In this context, human-mouse chimeras are a valuable tool for studying the behavior of iPSC-derived cell subtypes xenografted in murine models of AD (Balusu et al., 2023; Espuny-Camacho et al., 2017; Hasselmann et al., 2019; Mancuso et al., 2019). However, like with any experimental model, some trade-offs are to be expected. Human cell engraftment is performed in immune-deficient AD strains, thereby missing the critical contribution of the immune system to the generation of the pathological phenotype.

Complementing this, co-culture 3D systems and human brain organoids – multicellular, complex 3D structures – derived from iPSC provide a physiologically relevant platform to study the complexity of AD (Cenini et al., 2021; Kim et al., 2020; Penney et al., 2020). Of note, developing novel and user-friendly differentiation protocols for iPSCs into cells of the central nervous system is democratizing this technology, making it accessible to laboratories with limited expertise in stem cell research. This broadens the availability of iPSC-based approaches and facilitates the testing of disparate, non-mainstream hypotheses. Nevertheless, human cell- and organoid-based models possess inherent limitations. These include the absence of a fully developed and functional nervous system (i.e. complex circuit dynamics), the lack of tissue vascularization, and the inability to capture the intricate interplay between multiple organ systems (Andrews and Kriegstein, 2022).

To bridge these gaps, there is now mounting interest in larger animal models, including non-human primates (Jennings et al., 2016). These animal models still bear discrepancies in brain structure and function compared to humans, potentially affecting the translational capacity of the findings, let alone ethical concerns (Bailey and Taylor, 2016). Complementing this, a comparative biology approach has been recently proposed. This perspective stems from the idea that companion animals undergo concomitant age-related changes and share the same environment and lifestyle of the patients, thereby acting as a proxy of the complex network of factors that modulate the disease (de Sousa et al., 2023).

These diversified preclinical settings must be supplemented with human studies and clinical research data. In this regard, integrating in silico studies has great potential. One area revolves around developing multi-scale computational models, which provide an eco-system for integrating and interrogating molecular, cellular, and network-level interactions (Rollo et al., 2023). In silico work can also foster the development of personalized medicine approaches for AD, including factors acting inside and outside the CNS (Doraiswamy et al., 2018; Lee et al., 2019; Massetti et al., 2024). By incorporating individual patient data, including genetic profiles and environmental exposures, computational models can help predict disease progression and identify optimal treatment strategies tailored to each patient’s unique circumstances (Forloni, 2020). Finally, in silico analysis can be employed to explore the heterogeneity of AD cases, considering the diverse clinical presentations and progression patterns observed among patients.

### Conclusions

In conclusion, the limitations of current preclinical AD models and the questionable benefit observed in anti-Aβ clinical trials call for an urgent reconsideration of our strategies (Granzotto and Sensi, 2024; Høilund-Carlsen et al., 2023; Kepp et al., 2023b).

On the preclinical front, a more comprehensive setting involving other experimental systems and more rigorous experimental designs is required to guarantee the cost-effective generation of data with high translational value (Cozachenco et al., 2023).

A more nuanced and context-dependent experimental approach, taken with careful thought, is crucial for the development of effective disease models and, ultimately, for improving our ability to prevent, diagnose, and treat this devastating disorder.
